# A Novel Transgenic Sf9 Cell Line for Quick and Easy Virus Quantification

**DOI:** 10.3390/insects15090686

**Published:** 2024-09-11

**Authors:** Kyu-Seek Kim, Jun-Su Bae, Hyuk-Jin Moon, Do-Young Kim, Soo-Dong Woo

**Affiliations:** 1 Department of Agricultural Biology, College of Agriculture, Life & Environment Science, Chungbuk National University, Cheongju 28644, Republic of Korea; zpdlems@naver.com (K.-S.K.); bjs0942@naver.com (J.-S.B.); qlfmzks@chungbuk.ac.kr (H.-J.M.); un-joo6302@daum.net (D.-Y.K.); 2 IPBL Inc., Cheongju 28644, Republic of Korea

**Keywords:** baculovirus expression system, virus quantification, transgenic cell line, Sf9 cells, Sf9-QE cells

## Abstract

**Simple Summary:**

In the medical and pharmaceutical industries, insect cell lines are used to produce useful recombinant proteins, with their production necessitating the genetic engineering of recombinant insect viruses. Recombinant viruses can be used in experiments according to a virus quantification process. However, current virus quantification methods are complicated by their skill and experience requirements and long durations. In this study, we attempted to generate a novel transgenic insect cell line for the prompt and convenient quantification of insect viruses. By introducing a fluorescent gene, Sf9-QE, a transgenic insect cell line that rapidly emits high fluorescence in response to viral infection was generated. Using the Sf9-QE cell line, the quantification completion time was shortened by 4 to 6 days compared to existing insect cell lines, and convenient quantification was also facilitated by fluorescence photometry. The novel transgenic insect cell line generated, Sf9-QE, will be very useful in the production of and research on various medically and pharmaceutically applicable recombinant proteins, greatly contributing to human health and medical care.

**Abstract:**

The following study was conducted to generate a transgenic Sf9 cell line for rapid and easy virus quantification in the baculovirus expression system (BES). The hr3 (homologous region 3) and *39K* and *p10* promoters were used as the expression structures to induce rapid and intense expression of the enhanced green fluorescent protein gene in cells in response to viral infection. Of 20 transgenic Sf9 cell lines generated using the piggyBac system, the cell line that showed the highest fluorescence expression in the shortest time in response to viral infection was selected and named Sf9-QE. The average diameter of the Sf9-QE cells was around 16 μm, which is 2 μm smaller than the average diameter of Sf9 cells, whereas the rate of cell proliferation was around 1.6 times higher in the Sf9-QE cells. Virus quantification using the Sf9-QE cell line did not produce significantly different results compared to the other cell lines; however, the time required for complete virus quantification was approximately 5.3 to 6.0 days for the Sf9-QE cells, which is around 4 to 6 days shorter than the time required for the other cell lines, enabling convenient and accurate virus quantification via fluorescence photometry within around 6.0 to 6.3 days. The properties of the Sf9-QE cells were stable for up to at least 100 passages.

## 1. Introduction

The baculovirus expression system (BES) produces a variety of industrially useful recombinant proteins using baculoviruses and insect cell lines. The BES produces recombinant proteins from insects, which are eukaryotic organisms, and can produce a variety of proteins with high activity through various post-translational modification processes [1]. Since the BES enables the production of a target protein through the generation of a recombinant virus carrying the target gene, purification, proliferation, and titration of the virus are essential. Therefore, producing a recombinant protein using the BES generally takes at least around 4 to 8 weeks from the generation of the recombinant virus to the identification of the target protein. Specifically, the virus quantification process takes at least 1 to 10 days to complete, depending on the method.

Some of the commonly used virus quantification methods include plaque assays, TCID50 (tissue culture infection dose 50), qPCR (quantitative PCR), flow cytometry, and ELISA (enzyme-linked immunosorbent assay); however, each method has its limitations, such as the special equipment requirements and the difficulties encountered during the experimental process or problems pertaining to its accuracy [2,3,4,5,6]. Among these methods, the end-point dilution method involving TCID50 is the most commonly used approach, with a low error probability and the ability to quantify infectious viruses only through an all-or-none method [7]. However, the TCID50 method has a major disadvantage in that it is difficult for non-experts to accurately determine whether viral infection is present, and the results obtained also vary greatly depending on the individual performing the experiment. Therefore, to allow non-experts to easily identify viral infections, Sf9-ET, a transgenic Sf9 cell line that only expresses enhanced green fluorescent protein (EGFP) upon viral infection (thus allowing for effortless confirmation), was developed and commercialized thereafter [8]. Although viral infection can be easily checked using Sf9-ET cells, it has been reported that virus quantification using fluorescence photometry is difficult due to low fluorescence expression.

In the following study, we attempted to develop a transgenic Sf9 cell line with enhanced fluorescence expression that not only facilitates effortless virus quantification but also accomplishes this rapidly. For this purpose, a new transgenic cell line that expresses EGFP rapidly and intensely only upon virus infection was generated, and its characteristics, as well as the accuracy, speed, and convenience of virus quantification, were evaluated.

## 2. Materials and Methods

### 2.1. Cells and Viruses

The *Spodoptera frugiperda* cell line Sf9, the transgenic cell line Sf9-ET (Kerafast, Boston, MA, USA), and the transgenic cell lines generated in this study were maintained at 27 °C in SFM900 II medium (Gibco, Grand Island, NY, USA). The viruses used comprised rMultiBac, rAc-LacZ, and rAc-PPV-VP2. rMultiBac was generated using Multibac (Geneva Biotech, Geneva, Switzerland), a bacmid derived from *Autographa californica* nucleopolyhedrovirus (AcMNPV-E2; GenBank No. KM667940.1). rAc-LacZ and rAc-PPV-VP2 are recombinant viruses that express the lacZ gene of *Escherichia coli* and the VP2 gene of porcine parvovirus under the control of the *polyhedrin* (*polh*) promoter, respectively. Routine cell culture maintenance, cell counting, and virus propagation procedures were performed as previously reported [3]. The cell number, size, shape, and survival rate were determined using FACSCOPE^®®^ B (CURIOSIS, Seoul, Republic of Korea) [9]. Cell viability was checked after trypan blue staining.

### 2.2. Construction of the Transfer Vector

As a vector for virus-induced transient expression, pHIP-EGFP was constructed by cloning the EGFP gene into the pHIP vector, which consists of the hr3 (homologous region 3) and *IE1* and *p10* promoters derived from AcMNPV-E2 (Figure S1). The *polh* promoter and its burst sequence (BS) were PCR-amplified using AcMNPV-E2 DNA as a template (Table S1) and cloned into the pHIP-EGFP vector to create pAc-BS-EGFP. As additional promoters, the *p6.9*, *vp39*, and *39K* promoters were each PCR-amplified (Table S1) and cloned into pAc-BS-EGFP to produce pAc-p6.9-EGFP, pAc-VP39-EGFP, and pAc-39K-EGFP, respectively. The *p10* promoter from pHIP-EGFP was cloned into the aforementioned vectors to construct pAc-p10-EGFP, pAc-p6.9-p10-EGFP, pAc-VP39-p10-EGFP, and pAc-39K-p10-EGFP, respectively. The transfer vector for generating the transgenic cell lines was constructed using pPIGA3-neo, which contains the piggyBac transposon recognition-flanking regions and is capable of expressing the neomycin resistance gene. pPIG-Donor was constructed by cloning the final expression structure into the pPIGA3-neo vector (Figure S2). Lastly, the structures of all the vectors were confirmed through nucleotide sequence analysis (COSMOgenetech, Daejeon, Republic of Korea).

### 2.3. Virus-Inducible Transient Expression

For the intracellular transfer of virus-inducible transient vectors, 4 × 10^5^ cultured cells were used in a 12-well plate. According to the manufacturer’s instructions, 1.8 μg of DNA was transfected into the cells using Cellfectin II Reagent™ (Invitrogen, Carlsbad, CA, USA). At 16 h after DNA transfection, rMultiBac was inoculated with 0.01 MOI (multiplicity of infection), and the intensity of fluorescence expression was measured daily.

### 2.4. Generation of the Transgenic Cell Line

The transfer vector and helper vector pHAPIG [10] were mixed and transfected into the Sf9 cells. G418 (Invitrogen, Carlsbad, CA, USA) was added 2 days after transfection, and only cells that showed resistance and survived were isolated. For cell isolation, cell colonies formed from single cells were dissociated by pipetting and transferred to 96-well plates, respectively. Isolated cells that were stabilized through subculture of 10 passages in medium supplemented with the G418 antibiotic (800 μg/mL) were selected and used in the experiments. In the transgenic cells, the introduced expression cassette (from the hr3 to the EGFP gene) was confirmed by isolating the entire cell DNA and performing a PCR assay (Table S1).

### 2.5. Virus Titration

The virus titer was measured using the end-point dilution assay method. The cells were distributed in a 96-well plate at a concentration of 2.0 × 10^4^ cells/90 μL per well, and the virus culture was serially diluted to 10^−5^, 10^−6^, 10^−7^, and 10^−8^ times. Then, 10 μL of the virus inoculum at each dilution was inoculated into 8 wells. After this step, the cells were cultured at 27 °C, and the number of virus-infected and uninfected wells was examined each day using phase-contrast and fluorescence microscopes. In addition, for the EGFP-transformed cell lines, the number of virus-infected wells was additionally evaluated by measuring the fluorescence intensity. The virus titers were calculated in PFUs (plaque-forming units) from the TCID50 value.

### 2.6. Fluorescence Intensity Measurement

To measure the fluorescence intensity of EGFP, the virus-infected cells were collected at one-day intervals and washed with ice-cold PBS. Lysates were prepared by incubating the cells with 400 µL of lysis buffer (20 mM Tris-HCl, 500 mM NaCl, 1 mM EDTA, 0.1% Tween 20, and pH 7.0) and a protease inhibitor cocktail (Sigma-Aldrich, Burlington, MA, USA) for 30 min on ice. The background value was corrected using only the assay buffer. Fluorescence was measured at room temperature in 96-well plates with a minimum test volume of 100 µL. The fluorescence intensity of the resulting samples was measured using a Synergy HTX Plate Reader (BioTek Inc., Winooski, VT, USA) with an excitation filter of 488 nm and an emission filter of 510 nm. A minimum of three experiments were performed.

## 3. Results and Discussion

### 3.1. Optimal Expression Structure of the Marker Gene

**Figure 1:**
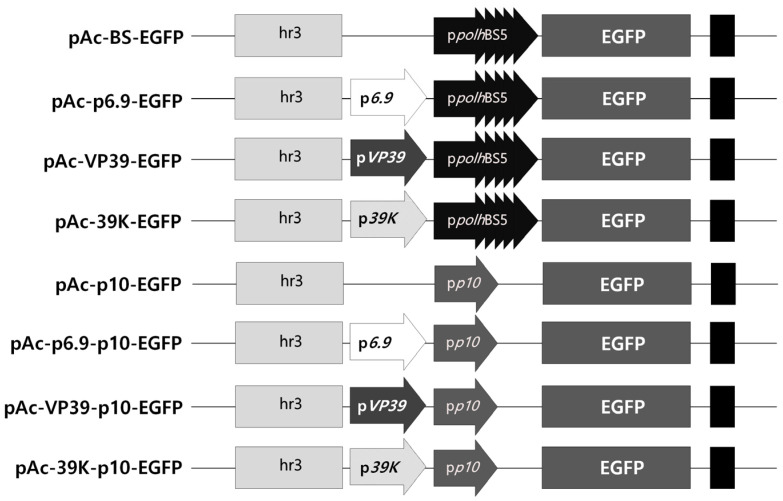
Schematic representation of the virus-induced transient expression vectors expressing EGFP. The very late *p10* or *polh* promoter with four repeated BSs was used as the main promoter to express EGFP. Additional promoters, such as *p6.9*, *vp39*, or *39K* promoters, were inserted between the main promoter and the transcription factor hr3.

**Figure 2:**
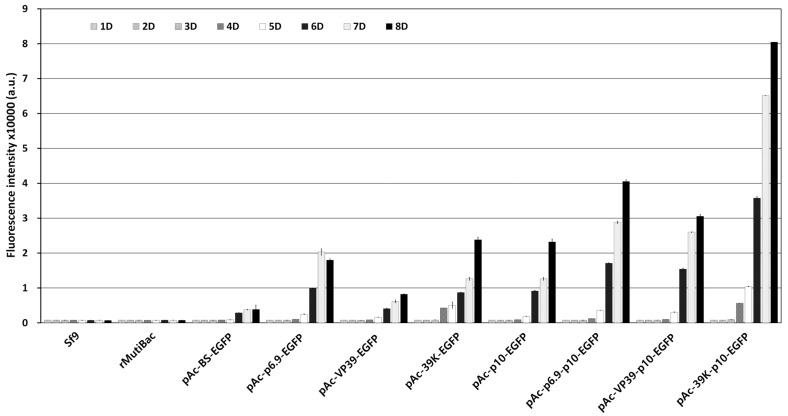
Fluorescence intensity of Sf9 cells by virus-induced transient expression. Each virus-induced transient expression vector was introduced into Sf9 cells and then inoculated with rMultiBac at an MOI of 0.01. The fluorescence intensity of the cell extracts was measured using fluorescence photometry up to 8 days p.i. and is shown in arbitrary units (a.u.). The data are expressed as the means ± standard error (SE). All experiments were replicated three times.

The optimal expression structure for rapid and intense expression of the intracellular marker genes upon viral infection only was determined. For virus-induced transient expression, the transcription factor hr3 is commonly used, and the *p10* or *polh* promoter with four repeated BSs was used as the main promoter (Figure 1). As additional promoters, *p6.9*, *vp39*, and *39K*, which were active earlier than the *p10* or *polh* promoters, were used. The expression of the EGFP gene under each promoter structure was incited by viral infection, and the fluorescence expression was compared and evaluated over time. Fluorescence expression as a response to virus infection was seen in all the vector structures, with the level of expression varying depending on the promoter structure (Figure S3). However, no fluorescence was observed in the absence of viral infection. These results indicated that the promoters used did not allow visible expression of EGFP without viral infection. The *p10* promoter induced higher expression as the main promoter than the *polh* promoter. Additionally, when an additional promoter was used, the expression was intensified, occurring 1 or 2 days earlier than in its absence. In comparing the fluorescence intensity, when the *p10* promoter was used as the main promoter, the expression observed was approximately 2.3 to 7.6 times higher than that with the *polh* promoter (Figure 2). Among the additional promoters, the *39K* promoter showed the greatest effect, exhibiting 2.5 to 5 times higher expression than the other additional promoters 5 days post-infection (p.i.). On this basis, the 39K-p10 promoter structure, which showed the fastest and highest level of virus-induced expression, was ultimately chosen as the promoter structure for transgenic cell line generation.

In addition to the main promoters, such as *polh* and *p10*, additional effects of the *p6.9*, *39K*, and *vp39* promoters on the *polh* promoter have previously been reported in the literature. For example, for the *polh* promoter, a hyperexpression vector was constructed whose expression efficiency was significantly increased following the addition of these promoters and transcription factors [11]. However, these researchers focused on enhancing expression with additional promoters in recombinant viruses, and thus far, there have been no reports of enhancement according to viral infection in plasmid DNA. In contrast, in our study, we were able to demonstrate the effect of enhancing expression through the inclusion of an additional promoter in plasmid DNA, although the effect differed slightly from that observed in recombinant viruses. In recombinant viruses, the *p6.9* promoter was shown to be the most effective. In contrast, in plasmid DNA, the *p6.9* promoter showed superior effectiveness until 7 days p.i., after which point the *39K* promoter was more effective. One of the biggest differences between recombinant virus DNA and plasmid DNA is that in recombinant viruses, the copy number of the target gene is greatly increased by virus proliferation; however, plasmid DNA does not replicate and thus does not increase in quantity. Interpreting any alterations in the expression efficiency between virus and plasmid DNA based on this difference in the copy number of the target gene alone would be difficult. In fact, the *39K* and *vp39* promoters have been more widely used in the generation of transgenic cells in previous studies, whereas the *p6.9* promoter has been more frequently used to increase the expression efficiency of recombinant viruses [11,12,13,14,15,16,17,18]. Therefore, it can be inferred that there will be some difference in promoter activity when these promoters are present or absent in the viral genome. In addition, in plasmid DNA, the *p10* promoter was associated with a higher expression efficiency than the *polh* promoter. Various studies have shown slightly different results regarding the expression strength of these promoters; in general, however, the *polh* promoter is more frequently used than the *p10* promoter [19,20,21]. Nevertheless, as previously reported in a study on a transactivation vector [22], the *p10* promoter is more efficient when used for plasmid DNA expression, such as in virus-induced transient expression. Our results concur with this finding. Overall, regardless of the use of an additional promoter, all the experiments showed higher expression when the *p10* promoter was the main promoter used. Further research will be required to elucidate the reasons for the differences in the promoter activity between virus and plasmid DNA according to various aspects of the system, including the transcription factors, activation time, and so on.

### 3.2. The Generation and Selection of the Transgenic Cell Lines

**Figure 3:**
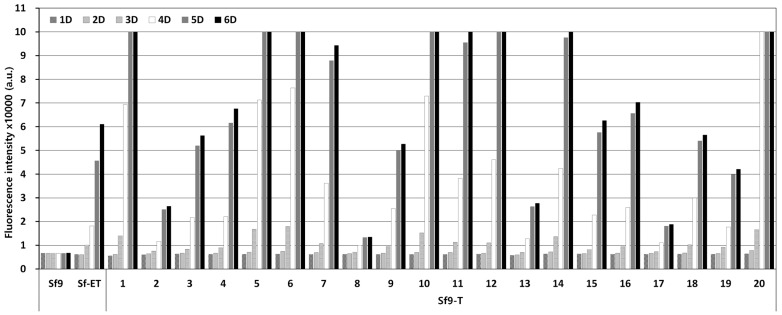
Fluorescence intensity of Sf9-T cells by virus infection. Cells were infected with rMultiBac at an MOI of 0.1. The fluorescence intensity of the cell extracts was measured using fluorescence photometry up to 6 days p.i. and is shown in arbitrary units (a.u.).

A DNA fragment capable of expressing EGFP under the final expression structure was transformed into the Sf9 cell genome using the piggyBac vector. After antibiotic treatment, cell colonies formed from a single cell were separated and cultured in a 96-well plate. After that, only surviving cells were passaged up to 10 times using 24-well and 6-well plates. Finally, 20 transgenic cell lines were selected from 92 initial cell colonies. Transgenic cell colonies were selected through antibiotic treatment and subcultured more than 10 times to ultimately select 20 transgenic cell lines. The selected transgenic cell lines were named Sf9-T, and PCR assay confirmed the presence of the EGFP gene in the cell genome. The fluorescence expression levels in response to virus infection were compared for the 20 selected Sf9-T cell lines. According to the results, clear fluorescence expression was observed from 3 days p.i. in all the Sf9-T cell lines, including Sf9-ET, which was used as a control (Figure 3). Of note, fluorescence expression was observed in the Sf9-T-20 cells at 1 day p.i. and had already reached its maximum at 4 days p.i, at which point the fluorescence intensity was approximately five times that in the Sf9-ET cells. Therefore, the Sf9-T-20 cell line, which showed the most rapid and intense fluorescence, was selected as the final transgenic cell line and named Sf9-QE (with “QE” standing for quick and easy).

The piggyBac system used to generate the transgenic cell lines in this study is a transformation technology that involves the use of the transposable element of the cabbage looper *Trichoplusia ni* [23,24]. The piggyBac transposase, a transfer enzyme, recognizes specific base sequences (inverted terminal repeat sequences (ITRs)) contained at both ends of the target gene and cuts them to transfer the target gene to the TTAA base sequence in the cell genome. When using this technology, there are no limitations on the length of the target gene, and it is recognized as an extremely useful genetic engineering tool that can be applied not only to insects but also to various vertebrates, including humans, enabling rapid gene transformation with high efficiency [25]. The copy number of the target gene introduced by the piggyBac system ranges from a single copy to hundreds of copies or more [26,27,28,29] and can be controlled according to the specific experimental conditions, with the concession, however, that it is hard to accurately control the copy number. Additionally, because the expression level of the target gene is proportional to the number of copies of the target gene introduced, the copy number introduced is an important factor in the gene expression. However, the introduction of a large number of copies of a target gene into the cell genome can alter the overall cell characteristics, including physiological characteristics, which can also significantly impact cell survival. Therefore, this complicates the preservation of the original cell characteristics upon the introduction of the target gene and the selection of transformed cell lines. Accordingly, before evaluating the suitability of the selected Sf9-QE cell line for virus quantification, the basic biological characteristics of the cells were investigated to determine whether they were changed by the introduction of the target gene and to examine whether they could be used for virus quantification purposes.

### 3.3. Characteristics of Sf9-QE

**Figure 4:**
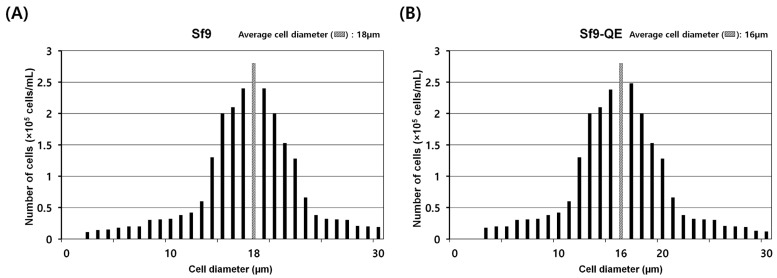
Diameters of Sf9 (**A**) and Sf9-QE (**B**) cells. Cell size was measured using FACSCOPE^®®^ B (CURIOSIS, Seoul, Republic of Korea). The hatched box indicates the median value.

**Figure 5:**
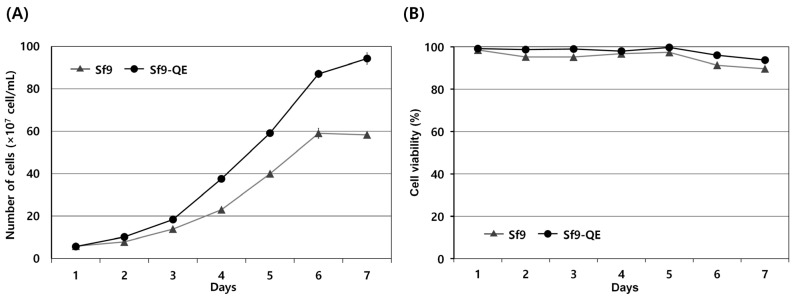
The cell proliferation growth curves (**A**) and viability (**B**) of Sf9 and Sf9-QE cell lines. Cell viability was determined through trypan blue staining. All experiments were replicated three times.

The external appearance of the Sf9-QE cells was no different from that of the Sf9 cells exhibiting epithelial morphology. However, their cell diameter, at approximately 16 μm on average, was approximately 2 μm smaller than that of the Sf9 cells, at approximately 18 μm in diameter (Figure 4 and Figure S4). When comparing cell proliferation, the Sf9-QE cells showed higher proliferation than the Sf9 cells from day 2 and had a proliferation rate around 1.6 times higher on day 7, when the highest rate of proliferation was exhibited (Figure 5A). However, no significant difference was found in the cell survival rate, which was found to be slightly higher for the Sf9-QE cells at an average of about 3% (Figure 5B). Additionally, no differences were observed between the two cell lines during the subculture process, including cell attachment or detachment to the culture plate. The results of our study suggest that the decreased size of the Sf9-QE cells may be related to their increased proliferation rate. However, the reasons for the decrease in cell size may be elucidated through further studies of the number and location of target genes introduced into the cell genome.

**Figure 6:**
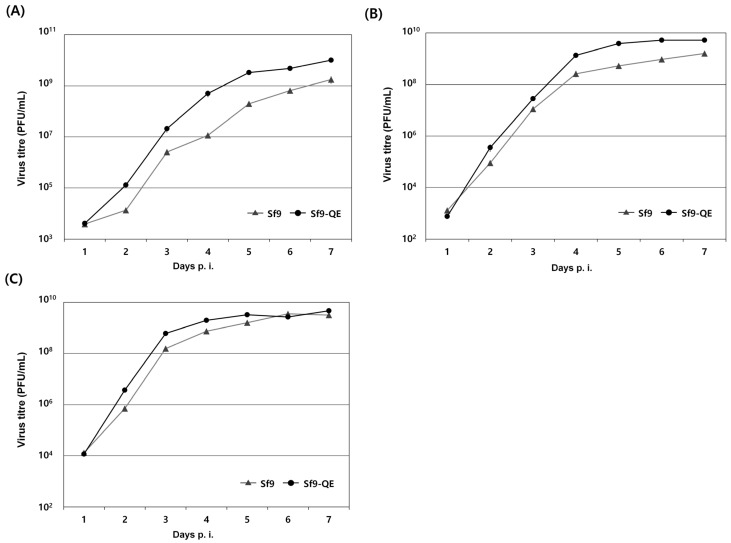
Virus growth curves in Sf9 and Sf9-QE cells. The cells were infected with rMultiBac (**A**), rAc-PPV-VP2 (**B**), and rAc-LacZ (**C**) at an MOI of 0.1. Supernatants were collected at the indicated day points, and the virus titers were determined by the end-point dilution assay method in Sf9 and Sf9-QE cells. The virus titer was calculated in PFU (plaque-forming units) from the TCID50 value. All experiments were replicated three times.

The viral susceptibility of the Sf9-QE cells, in which changes in cell size and the proliferation rate were shown, was evaluated using three types of viruses. Although there were some differences depending on the virus used, our results show that the Sf9-QE cells display slightly higher viral proliferation than the Sf9 cells from 2 days p.i. (Figure 6). Additionally, the virus yield in the Sf9-QE cells was approximately 1.5 times higher for rMultibac, approximately 3.2 times higher for rAc-PPV-VP2, and approximately 5.8 times higher for rAc-LacZ than it was for the Sf9 cells at 7 days p.i. The above results suggest that as the cell size decreases and the proliferation rate increases, viral proliferation also increases. This increased viral proliferation rate in the Sf9-QE cells is a positive result in the context of confirming viral infection more quickly. Therefore, we believe that the Sf9-QE cell line would be suitable for use in virus quantification procedures.

### 3.4. Suitability of the Sf9-QE Cells for Virus Quantification

**Figure 7:**
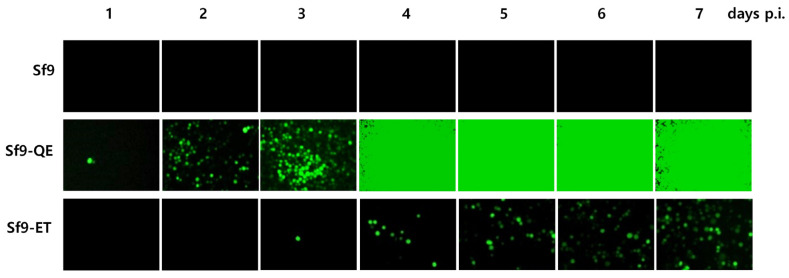
Fluorescence micrographs of Sf9-QE cells by virus infection. Cells were infected with rMultiBac at an MOI of 0.1.

The accuracy of virus quantification using Sf9-QE cells was evaluated by comparing them with the Sf9 and Sf9-ET cells using three types of viruses. As a result of our efforts, the quantitative values obtained were 1.2~4.3 × 10^7^ PFU/mL for rAc-LacZ, 6.9~7.3 × 10^8^ PFU/mL for rAc-PPV-VP2, and 2.7~8.0 × 10^8^ PFU/mL for rMultibac (Table 1). There were slight differences depending on the cell line used, although these were not significant. The completion of virus quantification took 11.3 to 12.0 days in the Sf9 cells, 5.3 to 6.0 days in the Sf9-QE cells, and 10.0 to 10.7 days in the Sf9-ET cells. The above results therefore confirm that virus quantification was possible in the Sf9-QE cell line at least 4 days and up to 6 days more quickly than in the other cell lines. Rapid virus quantification using the Sf9-QE cells was also confirmed as a possibility on the basis of the level of fluorescence caused by viral infection (Figure 7 and Figure S5). It is generally known that the time required for virus quantification in Sf9 cells is about 7 days. However, for highly accurate virus quantification, it may take more than 7 days. This is because the time required for completion of quantification may be longer when inoculating very low concentrations of viruses with high dilution ratios. In our study, quantification was performed for up to 12 days to more clearly compare the accuracy of virus titers between cell lines. In addition, single-cell infection was not scored in the judgment of virus infection wells. As a result, in Sf9 cells, virus quantification was completed in about 8 days at low virus dilution and in about 12 days at high dilution, which was the maximum observation period (Figure S6). In addition, in Sf9-ET cells, although previous reports reported that virus quantification was completed in 3 days [8], the results of our study showed that virus quantification was completed in about 4 days at low virus dilution and up to about 10 days at high virus dilution. On the other hand, in Sf9-QE cells, virus quantification was completed in about 3 days at low virus dilution and about 6 days at high dilution. Although the virus quantification completion time was somewhat longer as the observation time increased, fluorescent cells could be observed 3 days after virus infection in Sf9-ET cells and 1 day after infection in Sf9-QE cells (Figure 7), confirming that quantification was possible even in a shorter time. In addition, the finding that there was no significant difference in viral quantitation values between these cell lines suggested that viral quantitation values measured over a long period of time are more accurate.

To evaluate their convenience, the suitability of the Sf9-QE and Sf9-ET cells for virus quantification was evaluated using fluorescence photometry. Our results show no significant difference in the virus quantification values for either cell line when using microscopic observation or fluorescence photometry (Table 2). However, there was a significant difference between the two cell lines in terms of the time taken to complete virus quantification. Quantification was achieved using the Sf9-QE cell line in 6.0 to 6.3 days, while quantification using the Sf9-ET cells was completed in 12 days. Therefore, the above results confirm that while convenient virus quantification is possible using fluorescence photometry with both the Sf9-QE and Sf9-ET cell lines, the Sf9-QE cell line is much more time-efficient. To evaluate the stability of subculturing the Sf9-QE cells, the virus quantification ability was compared using cells subcultured 20 or 100 times after the establishment of the transgenic cell line. The results confirmed that the virus quantification ability of the Sf9-QE cell line did not significantly change at up to 100 passages (Table 3), suggesting that the characteristics of the Sf9-QE cell line can be stably maintained and proving its long-term utility.

To use recombinant viruses in the BES, accurate virus quantification is essential [30], guaranteeing the reliability of experimental results and quality control in industrial recombinant protein production. Therefore, in enhancing the convenience of the virus quantification process and dramatically shortening its duration, the Sf9-QE cell line could be a very useful cell line for both research and industry purposes. Although the Sf9-QE cell line established in the present study shows that faster quantification compared to the existing commercially available Sf9-ET cell line is possible, the results presented should be further clarified through additional studies that take the number of passages into consideration. However, the fact that the characteristics of the Sf9-QE cell line are very stably preserved at 100 passages indicates its utility. In conclusion, in facilitating rapid and convenient virus quantification, the Sf9-QE cell line is expected to be promising for research using the BES and in industry.

## Figures and Tables

**Table 1:** Virus quantification value and completion days for each cell line.

	Virus	rAc-LacZ		rAc-PPV-VP2		rMultiBac	
Cell Line		Titre (PFU/mL)	C. Days ^1^	Titre (PFU/mL)	C. Days	Titre (PFU/mL)	C. Days
Sf9		4.3 × 10^7^ ± 2.3%	12.0 ± 0.0	6.9 × 10^8^ ± 3.9%	12.0 ± 0.0	3.7 × 10^8^ ± 5.8%	11.3 ± 0.3
Sf9-QE		3.2 × 10^7^ ± 7.3%	5.7 ± 0.3	7.3 × 10^8^ ± 2.4%	6.0 ± 0.0	8.0 × 10^8^ ± 1.3%	5.3 ± 0.3
Sf9-ET		1.2 × 10^7^ ± 8.1%	10.7 ± 0.9	7.1 × 10^8^ ± 4.8%	10.0 ± 0.0	2.7 × 10^8^ ± 9.8%	10.0 ± 0.6

^1^ C. days: quantification completion days.

**Table 2:** Virus quantification value and completion days using fluorescence photometry.

	Virus	rAc-LacZ		rAc-PPV-VP2		rMultiBac	
Cell Line		Titre (PFU/mL)	C. Days ^1^	Titre (PFU/mL)	C. Days	Titre (PFU/mL)	C. Days
Sf9-QE	Microscope	3.2 × 10^7^ ± 6.3%	5.7 ± 0.3	7.3 × 10^8^ ± 3.4%	6.0 ± 0.0	8.0 × 10^8^ ± 2.3%	5.3 ± 0.3
	Photometry	3.1 × 10^7^ ± 7.3%	6.3 ± 0.3	7.2 × 10^8^ ± 2.4%	6.7 ± 0.3	7.9 × 10^8^ ± 1.3%	6.0 ± 0.3
Sf9-ET	Microscope	1.2 × 10^7^ ± 8.1%	10.7 ± 0.9	7.1 × 10^8^ ± 4.9%	10.0 ± 0.0	2.7 × 10^8^ ± 9.8%	10.0 ± 0.6
	Photometry	1.2 × 10^7^ ± 13.3%	12 ± 0.0	6.4 × 10^8^ ± 4.8%	12 ± 0.0	2.6 × 10^8^ ± 14.9%	12 ± 0.0

^1^ C. days: quantification completion days.

**Table 3:** Virus quantification value and completion days using 20 passages and 100 passages for Sf9-QE cells.

	Virus	rAc-LacZ		rAc-PPV-VP2		rMultiBac	
Passages		Titre (PFU/mL)	C. Days ^1^	Titre (PFU/mL)	C. Days	Titre (PFU/mL)	C. Days
P.20		7.9 × 10^8^ ± 3.7%	5.7 ± 0.3	6.9 × 10^8^ ± 6.1%	6.7 ± 0.3	8.3 × 10^8^ ± 5.3%	6.0 ± 0.0
P.100		7.3 × 10^8^ ± 3.1%	5.3 ± 0.3	7.1 × 10^8^ ± 6.9%	6.3 ± 0.3	8.4 × 10^8^ ± 5.1%	5.7 ± 0.3

^1^ C. days: quantification completion days.

## Data Availability

The original contributions presented in this study are included in the article/Supplementary Materials; further inquiries can be directed to the corresponding author.

## Supplementary Materials

The following supporting information can be downloaded at: https://www.mdpi.com/article/10.3390/insects15090686/s1. Table S1: Primers used in our experiment; Figure S1: Schematic diagram of virus-induced transient expression vectors expressing EGFP; Figure S2: Schematic diagram of the transfer vector for generating the transgenic cell lines; Figure S3: Fluorescence micrographs of Sf9 cells by virus-induced transient expression; Figure S4: Morphology of Sf9 and Sf9-QE cells; Figure S5: Fluorescence micrographs of Sf9-QE and Sf9-ET cells by virus infection on 5 days; Figure S6: Number of virus-infected wells over time in an endpoint dilution assay for virus quantification.
