# Supplementary material for: A Novel Transgenic Sf9 Cell Line for Quick and Easy Virus Quantification
Source: Insects. 2024 Sep 11;15(9):686. doi: 10.3390/insects15090686 (PMC11431869; doi:10.3390/insects15090686)
Supplement: Supplementary file 1 [file insects-15-00686-s001.zip › insects-3120005-supplementary.pdf]

Supplementary Data

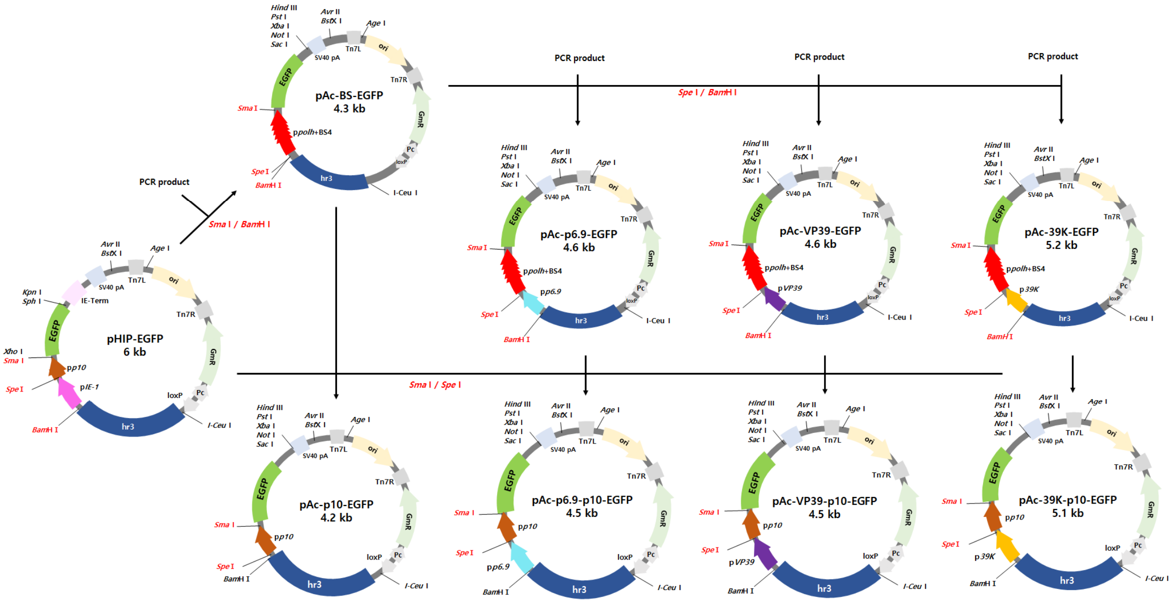

Figure S1. Schematic diagram of virus-induced transient expression vectors expressing EGFP.

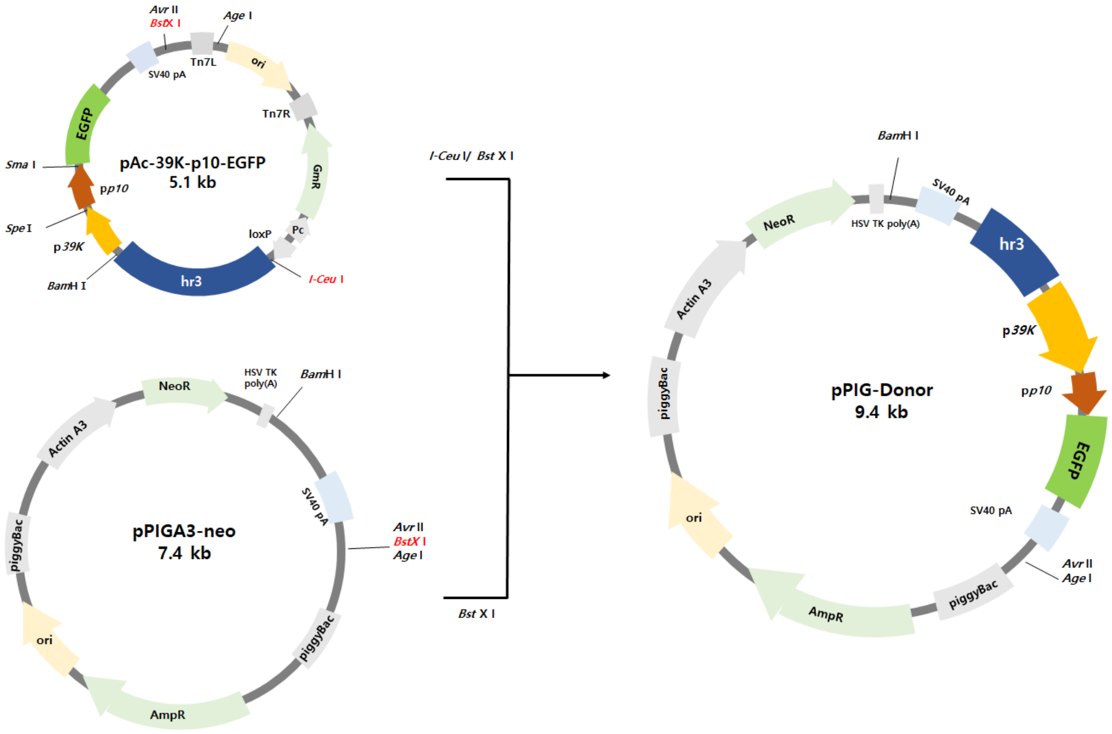

Figure S2. Schematic diagram of the transfer vector for generating transgenic cell lines.

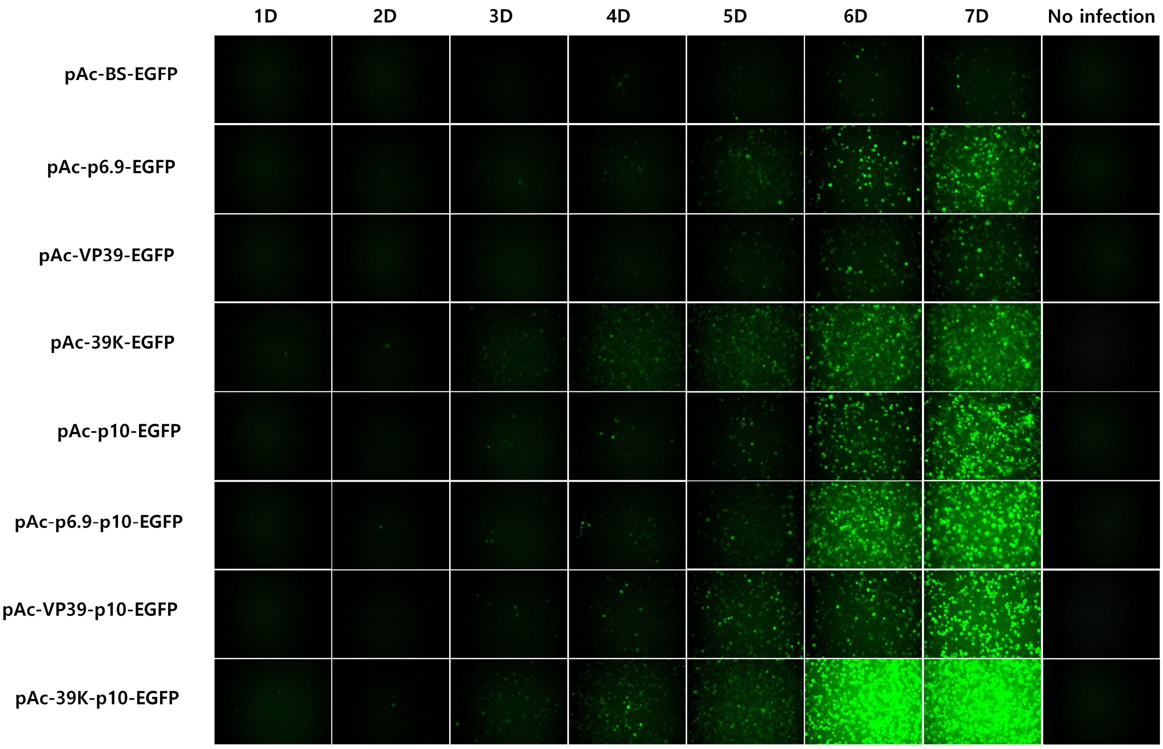

Figure S3. Fluorescence micrographs of Sf9 cells by virus-induced transient expression. Each virus-induced transient expression vector was introduced into Sf9 cells and then inoculated with rMultiBac at an MOI of 0.1. Micrographs observed on 7 days after introduction of each transient expression vector without viral infection were expressed as 'No infection'.

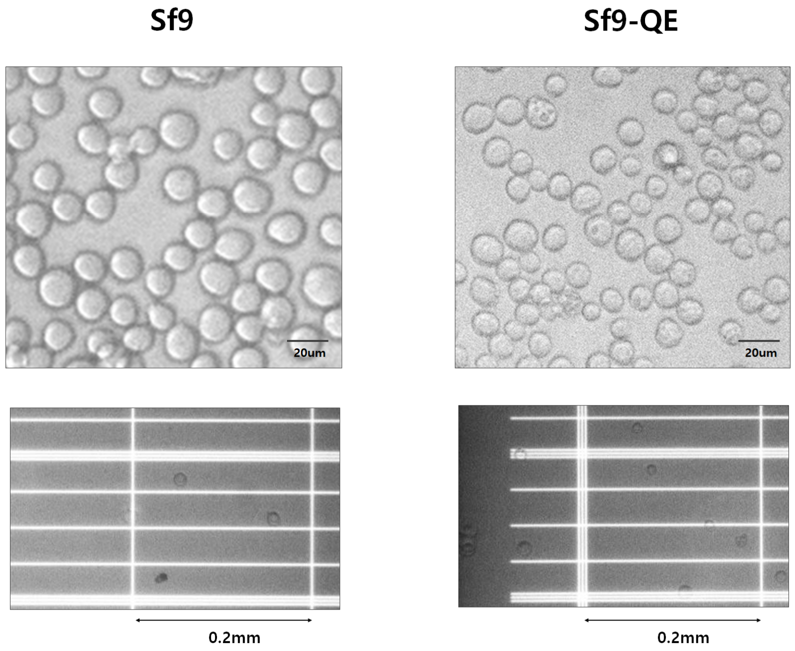

Figure S4. Morphology of Sf9 and Sf9-QE cells. Sf9-QE cells appear smaller than Sf9 cells on a hematocytometer (bottom).

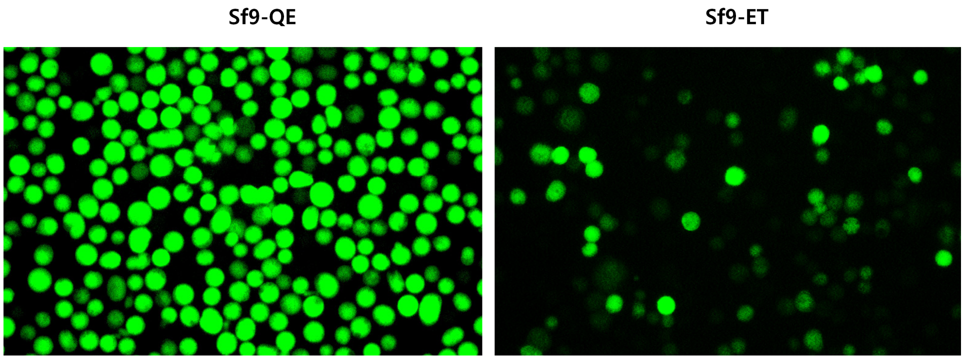

Figure S5. Fluorescence micrographs of Sf9-QE and Sf9-ET cells by virus infection on 5 days. Cells were infected with rMultiBac at an MOI of 0.1.

(A)

Sf9	1D	2D	3D	4D	5D	6D	7D	8D	9D	10D	11D	12D
10^{-5}	-	-	-	-	-	-	4	7	7	8	8	8
10^{-6}	-	-	-	-	-	-	-	2	2	2	2	3
10^{-7}	-	-	-	-	-	-	-	-	-	-	-	-
10^{-8}	-	-	-	-	-	-	-	-	-	-	-	-
Sf9-QE	1D	2D	3D	4D	5D	6D	7D	8D	9D	10D	11D	12D
10^{-5}	-	3	6	7	7	7	7	7	7	7	7	7
10^{-6}	-	1	2	2	2	3	3	3	3	3	3	3
10^{-7}	-	-	-	-	-	-	-	-	-	-	-	-
10^{-8}	-	-	-	-	-	-	-	-	-	-	-	-
Sf9-ET	1D	2D	3D	4D	5D	6D	7D	8D	9D	10D	11D	12D
10^{-5}	-	-	-	-	1	2	5	5	5	5	5	5
10^{-6}	-	-	-	-	-	-	-	-	-	-	1	1
10^{-7}	-	-	-	-	-	-	-	-	-	-	-	-
10^{-8}	-	-	-	-	-	-	-	-	-	-	-	-

(B)

Sf9	1D	2D	3D	4D	5D	6D	7D	8D	9D	10D	11D	12D
10^{-5}	-	-	1	2	4	7	8	8	8	8	8	8
10^{-6}	-	-	-	-	-	5	6	7	8	8	8	8
10^{-7}	-	-	-	-	-	1	1	3	3	3	3	4
10^{-8}	-	-	-	-	-	-	-	-	-	-	-	-
Sf9-QE	1D	2D	3D	4D	5D	6D	7D	8D	9D	10D	11D	12D
10^{-5}	1	3	6	8	8	8	8	8	8	8	8	8
10^{-6}	-	3	6	7	8	8	8	8	8	8	8	8
10^{-7}	-	1	3	3	3	4	4	4	4	4	4	4
10^{-8}	-	-	-	-	-	-	-	-	-	-	-	-
Sf9-ET	1D	2D	3D	4D	5D	6D	7D	8D	9D	10D	11D	12D
10^{-5}	-	-	2	5	8	8	8	8	8	8	8	8
10^{-6}	-	-	-	5	7	8	8	8	8	8	8	8
10^{-7}	-	-	-	1	2	3	3	3	3	4	4	4
10^{-8}	-	-	-	-	-	-	-	-	-	-	-	-

(C)

Sf9	1D	2D	3D	4D	5D	6D	7D	8D	9D	10D	11D	12D
10^{-5}	-	-	-	-	-	1	6	8	8	8	8	8
10^{-6}	-	-	-	-	-	-	1	6	7	7	7	7
10^{-7}	-	-	-	-	-	-	-	1	1	2	2	3
10^{-8}	-	-	-	-	-	-	-	-	-	-	-	-
Sf9-QE	1D	2D	3D	4D	5D	6D	7D	8D	9D	10D	11D	12D
10^{-5}	7	7	8	8	8	8	8	8	8	8	8	8
10^{-6}	7	7	8	8	8	8	8	8	8	8	8	8
10^{-7}	1	2	2	3	4	4	4	4	4	4	4	4
10^{-8}	-	-	-	-	-	-	-	-	-	-	-	-
Sf9-ET	1D	2D	3D	4D	5D	6D	7D	8D	9D	10D	11D	12D
10^{-5}	-	-	5	8	8	8	8	8	8	8	8	8
10^{-6}	-	-	3	6	7	7	7	7	7	7	7	7
10^{-7}	-	-	-	1	1	1	1	1	1	2	2	2
10^{-8}	-	-	-	-	-	-	-	-	-	-	-	-

Figure S6. Number of virus-infected wells over time in an endpoint dilution assay for virus quantification. Recombinant viruses rAc-LacZ (A), rAc-PPV-VP2 (B), and rMultiBac (C) were diluted from 10^{-5} to 10^{-8} times and inoculated into eight wells of a 96-well plate. The cumulative number of virus-infected wells over time at each dilution was counted. The red letters indicate the time at which virus quantification was finally completed. Final virus quantification values were determined from at least three replicates, and this figure shows an example of one of the results.

Table S1. Primers used in our experiment.

Usage	Primer[a]	Nucleotide sequences[b]
Vector construction	Ac-hr3-F	5' GATTTACGCGTAGAATTCTACTTGTAAAGCAAGTTAAAATAAG 3'
	Ac-hr3-R	5' GCTTTACGAGTAGAATTCTACTTGTAACGCATG 3'
	Ac-p10-F	5' GACCTTTAATTCAACCCAACAC 3'
	Ac-p10-R	5' GATTGTAAATAAAATGTAATTTACAGTATAG 3'
	Ac-pol-F	5' **GGATCC**ACTAGTATCATGGAGATAATTAAAATGATAACC 3'
	Ac-pol-R	5' **CCCGGG**ATTTATAGGTTTTTTATTACAAAACTG 3'
	Ac-polBS-F	5' CTGTTTTCGTAACAGTTTTG 3'
	Ac-polBS-R	5' ATTTATAGGTTTTTTATTACAAAACTG 3'
	Ac-vp39-F	5' **GGATCC**GTCTTGTAAGGCAGTTTGATTTC 3'
	Ac-vp39-R	5' **ACTAGT**ATTGTTGCCGTTATAAATATGG 3'
	Ac-p6.9-F	5' **GGATCC**AAATTCCGTTTTGCGACGATG 3'
	Ac-p6.9-R	5' **ACTAGT**GTTTAAATTGTGTAATTTATGTAGCTGTAATTTTTACC 3'
	Ac-39k-F	5' **GGATCC**AAGGCTGTCCTGCTGTG 3'
	Ac-39k-R	5' **ACTAGT**GTTTGCTTCTTGTAAACCTTTGAAACAAC 3'
Sf9-T check	EGFP-F	5' ATGGTGAGCAAGGGCGAGG 3'
	EGFP-R	5' TTACTTGTACAGCTCGTCCATGCCG 3'
	QE-hr3-F	5' CATGCCACTACTCGGTTCCG 3'

[a] For each structure, the forward primer (F) is present in the forward direction with respect to the coding strand and the reverse primer (R) is present in the reverse direction with respect to the coding strand.

[b] Restriction enzyme site shown in bold.
